# Enablers and barriers to effective parenting within the first 1000 days: an exploratory study of South African parents and primary caregivers in low socio-economic communities

**DOI:** 10.1186/s12889-022-13179-9

**Published:** 2022-04-20

**Authors:** Babatope O. Adebiyi, Tessa Goldschmidt, Fatiema Benjamin, Inge K. Sonn, Edna Rich, Nicolette V. Roman

**Affiliations:** Centre for Interdisciplinary Studies of Children, Families and Society, University of the Western Cape, Cape Town, South Africa

**Keywords:** First 1000 days, Parents, Primary caregivers, Effective parenting, Enablers, Barriers, Qualitative research, South Africa, Low socio-economic communities

## Abstract

**Background:**

The first 1000 days is the period between conception and a child’s second birthday. Globally, research on parenting is in an advanced stage, but parenting research focusing specifically on parenting in this developmental phase is limited in South Africa. Therefore, this study explores the enablers and barriers to effective parenting within the first 1000 days through the lens of parents and caregivers in low socio-economic communities.

**Methods:**

This study was conducted in communities in South Africa considered low socio-economic communities in the Western Cape Province of South Africa. An exploratory qualitative research design explored the enablers and barriers to effective parenting within the first 1000 days of life. Thirty participants were purposively selected and interviewed in this study. A semi-structured interview schedule was used for all interviews. The data were analysed using inductive thematic analysis.

**Results:**

Two main categories emerged (effective parenting enablers and effective parenting barriers) during the data analysis. The main enablers of effective parenting within the first 1000 days of life include a support system, healthy behaviours/environment, unemployment/job opportunities, religion, information/knowledge, and professional assistance. On the other hand, the main barriers to effective parenting were low socio-economic circumstances, environmental circumstances, lack of partner’s support, the negative impact of technology, and lack of access to services.

**Conclusion:**

Enablers that need to be promoted for effective parenting range from support systems to professional assistance for parents. Also, barriers that need to be removed for effective parenting range from low socio-economic circumstances to a lack of partner’s support for parents. This is because effective parenting is vital in improving developmental outcomes for children within the first 1000 days of life. Therefore, there is a need to develop policies and interventions to promote effective parenting within the first 1000 days in the communities.

## Background

The first 1000 days is the period between conception and a child’s second birthday [1–3]. It is the critical period for disease prevention [1] and the fundamental window of opportunity for a child’s development [2]. These first 1000 days are especially crucial for the most active period of neurological childhood brain development [3]. Children's experiences in the first 1000 days of life have major effects on child development and well-being [4]. Studies have shown that a child with early and typical physical, social, emotional, language and cognitive development has better health and educational outcomes in childhood, and later in life, as well as employment outcomes in adulthood [5–7]. This indicates that development during the first 1000 days of life significantly contributes to the life trajectory of individuals.

Poor maternal newborn and child health (MNCH) is considered a global public health burden. In 2019, it was estimated that 5.2 million children younger than five years die from mostly preventable or treatable causes [8]. Furthermore, in low- and middle-income countries, over 250 million children younger than five years old may not reach their developmental potential because of poverty and other adverse conditions [9, 10]. MNCH forms part of the United Nations’ Sustainable Development Goals (SDGs) three “Good Health and Well-being for all, at every stage of life”. One of the targets of the SDG three is to reduce the global neonatal mortality rate from 19 deaths per 1,000 live births to 12 deaths per live births and eliminate all deaths of children under-5 due to preventable causes [11]. One of the ways to address poor MNCH, and achieve the indicated targets, could be to focus on parenting, specifically, parenting practices, knowledge, and skill in the first 1000 days of life.

Parents play a crucial role in the development of children during the first 1000 days of life. Specifically, the quality of parenting is essential because it is the most important of all modifiable factors that influence the course of a child’s development [12, 13]. Parenting, educational support and adequate health nutrition are imperative to early childhood development outcomes, and potential attainment [14]. There is a link between parenting, educational support, and adequate health nutrition. That is, the parental responsibility includes providing early educational support, adequate health nutrition, early stimulation, and safety through their parenting practices. International organizations such as the World Bank, United Nations Children’s Emergency Fund (UNICEF), and World Health Organisation (WHO) have identified parenting education programmes as a priority for improving child development outcomes in low- and middle-income countries [15]. These organizations suggested the need to strengthen parenting skills, competencies, and practices.

Parenting, particularly effective parenting (parents’ abilities to nurture, connect, interact, and engage with their children for the latter to learn, and grow into successful adults), supports physical, emotional, social, and intellectual development from infancy to adulthood [16]. Parenting approaches include permissive, uninvolved, authoritarian, and authoritative parenting styles [17]. Research has shown that each parenting approach is associated with a particular developmental outcome for a child [18]. For example, the authoritative parenting style is associated with assertive and self-reliant children, and parents have high responsiveness and high demandingness. In the authoritarian style, parents are low on responsiveness and high on demandingness and this style is associated with disconnected and withdrawn behaviour in children. A style in which parents have high responsiveness and low demandingness is called the permissive style and is associated with low self-control and low self-reliance. Additionally, children are more likely to misbehave because parents have low responsiveness and low demandingness. This type of parenting style is called the uninvolved style.

Research has shown that parenting styles predict the quality of parent–child relationships [19]. Consequently, parent–child relationships may have short and long-term implications for positive mental health and well-being [20]. For example, a study that examined the relationship between perceived parent–child communication and children's well-being measured: depression, self-esteem, substance use, and school adjustment revealing the importance of open and transparent parent–child communication to enhance adolescent well-being [21]. Furthermore, effective parenting practices enable parents to help their children be safe, be successful in many areas of their lives, including but not limited to emotional, behavioral, cognitive, and social, and stay physically, and mentally healthy[16]. Conversely, poor parenting practices have been reported to be associated with negative emotional outcomes in children [22]. Also, parents with a poor understanding of children's development are less likely to identify developmental delays in their children [23].

Sander and Turner [13], reported various enablers and barriers to effective parenting. The enablers and barriers are categorised into five areas: parental concerns, motivation, programme features, cognitions/affect, and social influences. For example, living in a stable and supportive home that is less stressful, having family members who are regularly employed, secure housing, and extended family and social support are conducive to good parenting [13, 24, 25]. On the other hand, stressful life circumstances such as violence (for example, intimate partner, domestic or community violence), living with someone with serious mental health issues, substance abuse, chronic physical health problems, homelessness, or involvement with the criminal justice system are not conducive to good parenting. Stressful situations reduce parental self-efficacy and parents’ capacity to support their children [26].

This study focuses on the first 1000 days of life because of the importance of this period to the development of children later in life. Globally, extensive research on parenting has been conducted; however, there is a paucity of research focusing specifically on parenting in the first 1000 days within South Africa. Studies on parenting in South Africa have examined certain aspects such as parenting styles [27, 28], parents' influence on child development [29], the father-son relationship, and child homicide [30, 31]. However, no study has explored the enablers and barriers to effective parenting in the first 1000 days of life. Therefore, this study explores the enablers and barriers to effective parenting through the lens of parents and caregivers in low socio-economic communities.

## Methods

### Study setting

This study was conducted in communities in South Africa considered low socio-economic communities namely Khayelitsha, Saldanha, Caledon, Mitchells Plain, Manenberg, Grabouw, Fisantekraal, Vredenburg, Genadendal, and Lamberts Bay. Population sizes and the number of households, as established by the 2011 South African Census, for each community are presented in Table 1 below [32]. The above-mentioned communities are located in the Western Cape Province of South Africa. These communities have similar characteristics, which include ethnically diverse residents, high levels of poverty and unemployment, poor service provision, and low-income households [33]. The communities were selected because of the aforementioned characteristics, which can impact children's developmental outcomes within the first 1000 days. Also, the communities met the predetermined criteria for inclusion in our study.

**Table 1:** Population size and number of households per community

	**Community**	**Population size**	**Number of households**
1	Khayelitsha	391 749	118 809
2	Saldanha	99 193	28 835
3	Caledon	13 020	3 544
4	Mitchell’s Plain	310 485	67 993
5	Manenberg	61 615	12 834
6	Grabouw	30 337	7 708
7	Fisantekraal	12 369	3 712
8	Vredenberg	38 382	11 557
9	Genadendal	5 663	1 593
10	Lambert’s Bay	6 120	1 710

### Study design

This study used a qualitative research method to understand the perspectives of parents and caregivers on the enablers and barriers to the parenting of a child within the first 1000 days of life. Also, this study used an exploratory design, which is used when there is limited available literature on the topic or population being studied, to ultimately gain a deeper understanding of the phenomenon [34].

### Sampling procedure

A purposive sampling approach was used to recruit the target participant based on the following inclusion criteria: (1) participants were selected if they were parents or primary caregivers of children between ages of zero to two years, including pregnant women; (2) participants were included if they could speak and understand English, Afrikaans or isiXhosa; (3) and if they lived in low socio-economic communities of the Western Cape. Also, the exclusion criteria include the following: (1) participants were not selected if they were not parents or primary caregivers of children between the ages of zero to two years; (2) participants were not included if they could not speak and understand English, Afrikaans or isiXhosa; (3) and if they not lived in low socio-economic communities of the Western Cape. The research areas were purposely selected by the research team (low socio-economic communities). In the communities, the research team approached via face-to-face and selected potential participants based on the inclusion criteria. After selection, if they were willing to participate, they were subsequently interviewed.

### Ethical considerations.

This study was approved by the Humanities and Social Sciences Research Ethics Committee of the University of the Western Cape (HS17/6/15). The principles of ethics were followed in conducting this study. First, the study aim was explained to the potential participants. They were then informed that participation is voluntary and that they can withdraw at any time if they wish to do so, without any penalty. Participants were given an information sheet written in the language they understand (English, Afrikaans or isiXhosa). The information sheet contained the roles of participants in the research. The participants were asked to read and ask questions if necessary. Participants who agreed to participate after reading the information sheet were requested to sign a consent form. All the information obtained from participants were kept strictly confidential on a password protected computer known only to the research team. Also, pseudonyms were used throughout the manuscript to keep participants anonymous.

### Data collection

Semi-structured interviews were conducted by two female social science researchers from the research team, after consent were obtained from the participants. The one-on-one interviews were conducted in English, Afrikaans, and isiXhosa, depending on the language a participant understands and speaks. The interviews were conducted in the communities. An interview schedule was used to guide each interview, which lasted for about 30–60 min. The participants were asked various questions related to enablers and barriers to parenting, care, and support of a child within the first 1000 days of life. Questions on the interview schedule (Table 2) were used to start the interviews, and follow-up questions were used to probe for additional information, when necessary. After obtaining permission from the participants, all the interviews were audio-recorded. The data collection continued to theoretical saturation. The theoretical saturation was achieved when no ‘new’ information or insight was elicited from the participant, and no new themes were identified [35]. Overall, thirty participants were interviewed.

**Table 2:** Interview schedule

Examples of Questions
- What prevents parents from showing care and support to their children?
- Any obstacles that they think of that impact or affect the parenting of their children?
- What has been the most difficult part of parenting for you?
- What makes it hard for you to support and care for your child?
- What role does the father of your child play in your child’s life?
- What enables parents to show care and support for their children?
- What would help you to be a better parent than what you are now?
- What makes it easy for you to support and care for your child?
- What can affect your abilities to nurture, connect, interact and engage with their children?

### Data analysis

The inductive thematic analysis was used to analyse the data [36]. The inductive approach was used to generate the two main categories and various themes. Also, using an inductive approach, the audio-recorded interviews were transcribed verbatim. The interviews that were conducted in other languages were translated into English, following transcription. After the transcription and translation had been done, the two social science researchers from the research team, independently analysed the data and later combined their results. The coding discrepancies that occurred during the coding process were resolved through discussion. The two researchers read the transcripts repeatedly for familiarisation, derivation of meaning, and generation of initial codes. The purpose of coding is to reduce raw data into manageable size and parts that are relevant to the research question. The initial codes were organised to generate the final codes. Codes with similar ideas were clustered together to form sub-themes, and those sub-themes with similar ideas were further congregated to form the final themes. Finally, the themes were defined and supported by extracts from the transcripts.

### Trustworthiness and rigor of the study

In this study, credibility, transferability, dependability, conformability, and a reflexive approach to the inquiry and analysis were used to establish rigor and trustworthiness [37]. A detailed description of the study’s site, participants, and procedures used to collect data was provided to ensure transferability. To ensure dependability, a detailed description of methods of data collection, analysis, and interpretation was provided. In addition, transcripts were coded by two researchers of the research team independently, and they met afterward to discuss the findings. The discrepancies during data analysis were resolved through a consensus. Moreover, a single interview schedule was used to guide all the interviews. The interviewers conducted a member-checking, which is a recap of key points that originated from the interviews, to ensure credibility. A reflective journal is a document that contains the discussions, deliberations, and decisions made by the research team during the research processes. This journal was kept as part of the audit trail. In addition to the audit trail, verbatim transcripts of the participant's responses to questions were included in this manuscript, to ensure confirmability. In reporting this study, all the relevant aspects of the criteria for reporting qualitative research (COREQ) outlined by Tong, Sainsbury and Craig [38] were followed.

## Results

Table 3 provides information about the selected participants. In all, 30 participants were interviewed for this study. The majority of respondents were females (ages between 16 and 30 years) with an educational level of secondary school. In addition, the 30 participants include 28 parents and two grandparents, specifically grandmothers.

**Table 3:** Study participants

Characteristics	Participants (30)
**Gender**	
Female	29
Male	1
**Age (years)**	
16–30	17
31–45	9
46–60	3
61–75	1
**Highest of Level Education**	
Never Attend school	2
Grade (0–7)	2
Grade (8–11)	20
Grade (12)	4
Diploma	1
Degree	1
**Gender of Child**	
Unknown	1
Male	13
Female	16
**Age of Child (months)**	
0–6	6
7–12	11
13–18	2
19- 24	11

## Presentation of the categories and themes

The two main categories and various themes obtained during the data analysis are presented as follows:

### Category 1: effective parenting enablers

Effective parenting refers to the parents’ abilities to nurture, connect, interact and engage with their children for the latter to learn and grow into successful adults. In this study, enablers are factors that may allow parents to be more effective in their parenting roles. This category consists of numerous themes which highlight various factors that could enable parents to perform their roles of caring forand supporting children within the first 1000 days. These are presented in the identified themes, namely (1) support system—comprising family, partner, and community support; (2) healthy behaviours/environment; (3) unemployment/job opportunities; (4) religion; (5) information/knowledge, and (6) professional assistance.

#### Theme 1: support system

This theme encapsulates the importance of the support that parents receive either from their partner (or spouse), family members or the community as an enabler to good parenting. Support from family members such as parents was very common within the data. Also, grandparents of the children were the main source of support for parents either in the form of guidance, physical (childcare), or financial assistance.

##### Family

The support may be from their immediate families, such as mothers and fathers. This is illustrated in the quotations below.

“I will ask my mother for help or advice about what I should do in a situation if she gets seriously sick or seriously hurt. Then my mother will come and show me what I must do because my mother is the closest to me” (Participant 1, female, 24 years old).

“Probably when she was born [was the hardest]. That time because it was very difficult. Because my parents did not want me to have a child and they did not support me but afterward when she started growing up they started supporting me” (Participant 18, female, 19 years old).

From the extracts above, it was obvious that grandparents, particularly grandmothers, are very important in the quality of care children will receive from their parents. This type of support from grandmothers is primarily essential for young and first-time parents. This is because there are things a young and first-time parent may not know about caring for a child. These grandmothers are usually available to provide support for their children to take proper care of their grandchildren.

##### Partner

Partner is another form of support system expressed by some participants as a good parenting enabler. This is what the participants have to say.

“If you have a good support system you know, like your family, or the child’s father’s, or if they support you, and everything doesn’t come from yourself, then it’s actually easier” (Participant 11, female, 23 years old).

“No, because mmhhhh there is always help, there is always (smiles). The father is there, there are always the grandparents there showering with love and gifts and always providing without me even having to shout” (Participant 4, female, 30 years old).

Some of the participants highlighted above the role of partners (fathers). They described partners as a source of a good support system. They acknowledged that mothers require help to be effective in their parenting roles. However, the mothers believed that support from fathers will make it easier for them to nurture their children.

##### Community support

Individuals living together make up a family, and many families make up a society. Also, a section of a society can be referred to as a community. Therefore, community support is vital in the care and support of a child within the first 1000 days. The below extracts were expressed by some of the participants.

“There was always a lady who offered that type of programmes, the pregnancy classes she had. Then she would teach you every week, or every two weeks something different. And then they would do exercises. And if one of the members in the group was nearing her date of giving birth, then they would have a baby shower for the child and everything. It was nice” (Participant 11, female, 23 years old).

“I feel like more interactive stuff with other mommies there with you. You feel like you can relate, and there are things you can learn from other parents that you don’t necessarily learn anywhere else, you know what I mean” (Participant 14, female, 25 years old).

The statements above show the importance of community support. The mothers highlighted community support in the form of parenting programmes. During the programme, parents are taught various topics about parenting—how to take care of themselves and their children. There is also an opportunity for them to interact and learn from other parents in the community.

#### Theme 2: healthy behaviours/environment

This theme delineates the importance of healthy behaviours/environments for parents to better development outcomes within the first 1000 days of life. Parents believe healthy behaviours/environments during and after pregnancy will ensure their children’s health. Some of the participants said:

“Not to smoke, not to drink, look after your body and make sure that you take the tablets like now, for example, that you get at the clinic, because it helps you” (Participant 3, female, 20 years old).

“I ensure that I eat properly, that I drink my medication like my iron tablets, calcium tablets, other tablets and then, that it is just to ensure that I remain healthy” (Participant 11, female, 23 years old).

Also, the environment is an essential enabler for effective parenting. This is expressed by the statement below.

“Don’t smoke around the baby or smoke in the house if it is now a newborn baby and Uhm, don’t also argue or fight in front of the baby” (Participant 3, female, 20 years old).

The quotations above show that some of the parents believed that their behaviours and environments in which they live are important to their children's development. Also, behaviours and environments may affect parents’ abilities to raise a child effectively. They believe that they need to be careful about what they eat, drink and do. The quotes show that the parents are aware of the impacts of negative behaviours on their children.

#### Theme 3: employment/job opportunities

Some of the participants in the study attested that having a job or any other opportunity that could generate an income will enable them to perform their functions as parents better. Some of the participants said:

“Uhm, if I can get a job and like now work again, earn a salary, that will {make} me a better parent” (Participant 5, female, 20 years old).

“Not being dependent on other people, like getting clothes and stuff from people for my children, I want to get it for them myself” (Participant 12, female, 38 years old).

The above extracts show the importance of having a job for parents. They believe that with the ability to earn, they will be able to provide the basic necessities such as food, cloth, and other things for their children. In addition, they believe that earning capability will make them be less dependent on others for their children’s needs.

#### Theme 4: religion

A few participants believe religion has a role to play to be able to perform their functions as parents. For example, this is what a participant said:

“I should be more religious, that would help me a lot. Like doing my salaah’s (prayers), going to the mosque (a Muslim place of worship), investing all of my days – all of my life basically in doing what is expected of me as a Muslim” (Participant 15, female, 23 years old).

A few parents believe that being religious—obeying God’s teachings will make them a good parent. They believe God will give them wisdom and necessary things for them to take care of their children.

#### Theme 5: information/knowledge

This theme represents parents’ agreement that having information/knowledge in the form of parenting preparedness training will enable them to support their children effectively. In addition, parents would like information such as basic post-birth care. Some of the participants expressed the below extracts.

“How to look after the baby. how to care for her, to love her and to communicate with her and how to discipline her” (participant 3, female, 20 years).

“I will say more about caring for the child from birth. Not only about the child, but yourself also. How you, how you must handle the child in the first few days, and uhm, general stuff like, uhm when the child makes some signs, when you must, when you must look out for these signs and these signs and these signs, then you must know your child is now, need this now, the child needs that now. So, the small sicknesses that they [parent] have, the children sicknesses that they pick up, pick up so easily” (Participant 11, female, 23 years old).

“The information of how do you build a foundation of what you want your child to be” (Participant 25, female, 30 years old).

The participants’ comments show the importance of knowledge of childcare, such as how to love, communicate, discipline, and handle a child when the child needs something or is sick, and so on. They believe that having this knowledge will make them be able to nurture, connect, interact, and engage with their children.

#### Theme 6: professional assistance

A few participants see professional assistance from psychologists and social workers as enablers for effective parenting. They enumerated that professionals such as psychologists can teach parents how to take care of their children's emotional needs as well as their own needs. This is illustrated in the quotation below.

“Uhm, I would definitely need a psychologist on board to help in us understanding why your child behaves the way your child behaves, how you can help them and how you can uhm control uhm, on how you can handle whatever mood they in and basically understand why they doing what they doing and you teach and give you the tools on how to react to whatever mood they in so you don’t you know breakdown your child and stuff like that” (Participant 15, female 23 years old).

The above quote shows that assistance from professionals is required to take care of a child effectively. For a mother to properly care for a child, it requires understanding a child's moods, needs and behaviours. The participant mentioned psychologists, which means mothers understand the role of psychologists in understanding children's behaviours.

### Category 2: effective parenting barriers

In this study, barriers are factors that may prevent parents from being effective in their parenting roles. This category considers some of the challenges parents experience in their daily lives. These challenges present barriers to effective parenting practices within the first 1000 days. The following four themes (each with various components) were identified within this category: low socio-economic circumstances, environmental circumstances, lack of partner’s support, the negative impact of technology, and lack of access to services. These are discussed in more detail below.

#### Theme 1: low socio-economic circumstances

Participants narrated how low socio-economic circumstances could be barriers to effective parenting. Two circumstances were specifically highlighted by participants, namely unemployment and financial constraints.

##### Unemployment

Unemployment is a crucial factor when parents consider the challenges in their lives as they report that they cannot provide for their children as they would like to. Unfortunately, some of the parents seem to equate employment with being a better parent. Unemployment as a low socio-economic circumstance is illustrated in the quotations below**.**

“Uhm, unemployment, like now, makes it difficult for me. No, I’m not working” (Participant 5, female, 20 years old).

“There is nothing that would make me happy as much as I would if I would work, when I don’t work I become stressed” (Participant 30, female, 42 years old).

“If I can, like, get a job and like now work again, earn a salary, that will {make} me a better parent” (Participant 3, female, 20 years).

From the above quotes, it was clear that lack of a job can makes it difficult for parents to support and care for their children. This is because unemployment can make parents unhappy and become stressed. Stress can disrupt mood and affect parents’ behaviours towards their children. The disrupted mood may prevent parents from having proper interaction and engagement with their children.

##### Financial difficulties

Participants expressed an array of financial difficulties detrimental to their ability to care for their children effectively. For example, some of the participants said:

“Yes, there is one thing they want right now and I cannot give it to them. I said they have to wait two months (laughs) because the budget is tight and we have other plans so I can’t, what they want I can’t give it to them right now. So, I do feel bad” (Participant 4, female, 30 years old).

“It [child support grant] helps for his milk and Kimbie's (diapers) and it's not a lot. So, it’s just enough for his milk and Kimbie's” (Participant 9, female, 26 years old).

“I borrowed money for Kimbie's (diapers) and to buy her juice for school” (Participant 12, female, 38 years old).

The above statements show the negative impacts of being in financial difficulties. The parents indicated that they were unable to provide the basic needs of their children. Some participants indicated that they often have to borrow money from family or community members to provide for their children's needs.

#### Theme 2: environmental circumstances

This theme emerged as a result of parents' concern about the safety of their children outside of the home environment and reporting on unconducive home environments. Parents fear that their children will either get hurt by others and/ or be kidnapped.

##### Unsafe environment

Safety issues were mentioned by participants, as they feared that their children may be harmed outside their homes. From the quotes, they narrated that their children are susceptible to physical harm, sexual assault, and abduction. Therefore, parents prefer to be in close proximity to their children to protect them from harm. However, being in close proximity to their children may prevent parents from carrying out other daily activities beneficial to child development. Below are what participants said:

“The people come from the road or from somewhere and then they fight with bottles and they fight in front of you. Doesn’t matter if you’re walking in the road then they throw bottles your way. And it actually isn’t safe for me to sit in the yard with her. Something can just happen when they want to come and run in the yard” (Participant 1, female, 24 years old).

“These days the children disappear and the children get raped and the children get assaulted” (Participant 1, female, 24 years old).

“I raised her on my arm because I feel that she is safer on my arm. Because if she gets hurt over there, then I’m not close enough, but if she is on my arm, then she is safe” (Participant 12, female, 38 years old).

##### Unconducive home environment

The below statement shows that a lack of home space is a barrier to effective parenting of children within the first 1000 days. This is because children need an adequate space to play and develop. Children also need to breathe fresh air, but a small room may not be able to provide this because of a lack of space. This is reported in the quote below.

“The only thing that is affecting me right now is that I don’t have my own home, so mmmhhhh my room is small, so I need to provide them with a bigger space” (Participant 4, female, 30 years old).

#### Theme 3: lack of partner’s support

##### Family structure

The quotation described below shows that family structure, particularly being a single parent, was reported as a barrier to parenting. The participant narrated that being a single parent made it difficult to care and support for children effectively. She said:

“When I was a single parent for seven years. It was the most difficult because I was…… it was only me. With the help of grandparents but still, then eventually someone came along and….” (Participant 4, female, 30 years old).

##### Partner

It is difficult to care for and support a child when the father is not supportive. A participant said:

“I don’t have work and I struggle and her daddy does not give me any money” (Participant 23, female, 19 years old).

For parents to be effective in their parenting role, support from the father is needed for the provision of essential needs of children. The above extract from a participant shows that the father of the child is not supportive—the father does not support the mother with money.

#### Theme 4: negative impact of technology

Excessive access and use of technology devices can be a barrier to effective parenting. For example, a parent mentioned how technology can take her attention away from her child and that disciplining a child can be challenging when someone spends much time accessing technological devices. This is illustrated in the quotations below.

“Terrible this is actually a thing. Hey, I’m actually struggling with the discipline aspect. Sometimes you get carried away like me especially with social media, I tend to be on my phone and then I forget my child is actually around me. So, yeah. I need to be more attentive” (Participant 14, female, 25 years old).

The above-outlined quotation shows how social media takes her attention away from her child. Social media can be a source of information; however, it can be detrimental when someone spends a lot of time on it. Therefore, spending much time on social media can affect parents’ ability to train their children.

#### Theme 5: inadequate preparation for parenthood

Some young parents were not prepared for parenting, especially with the first child. This is expressed in the quotation below.

“With my first child, I was young and knew nothing. I didn’t even understand what was meant by pregnancy…then with my second I was married then but still I wasn’t prepared” (Participant 18, female, 19 years old).

The above statement shows that many parents, especially young parents, do not have knowledge on pregnancy and child care. This lack of knowledge may affect parents’ ability to nurture their children. This is because it requires a lot of childcare experience and preparation to effectively care and support a child.

#### Theme 6: lack of access to services

Access to services was also reported as a barrier to effective parenting. This is illustrated in the extract below.

“I would definitely need a psychologist on board to help us understand why your child behaves the way your child behaves” (Participant 29, female, 23 years old).

The above quote shows a lack of access to services, particularly professional services—those knowledgeable on how to care for a child could act as a barrier to effective parenting. This is because parents, especially first-time parents, may not understand why a child behaves in a particular way. Therefore, the inability of parents to understand a child's behaviour may affect their interactions and engagements with their children.

## Discussion

This study explored enablers and barriers to effective parenting within the first 1000 days of life within a South African context. The enablers of effective parenting within the first 1000 days of life included having a support system, healthy behaviours/environment, employment/job opportunities, religion, information/knowledge and professional assistance. The study yielded the following barriers: low socio-economic circumstances, environmental circumstances, lack of partner’s support, the negative impact of technology, and lack of access to services. Studies have shown that over 250 million children under five years old may not reach their developmental potentials in low- and middle-income countries for various reasons [9, 10]. Therefore, understanding the enablers and barriers that contribute to parenting within a South African context allows for favourable developmental outcomes for children, families, and communities. Lee Vygotsky [39] theorised that development and learning occur within the child’s immediate social context [40]. Parents and caregivers form part of a child’s first and most important social context [41]. Therefore, the parent–child relationship may be fundamental to early childhood developmental outcomes [42, 43]. There are many factors that have an influence on child development, especially during the first 1000 days. These factors include maternal and child health, socio-economic status, responsive caregiving, safety and security, early stimulation, safety and security for the parents, and the child and parenting knowledge and practices [9, 42, 44]. Thus, events and circumstances from as early as the first 1000 days of life may have a positive or negative impact on early child development outcomes and impact on the child’s schooling career. Many factors determine what parenting style a parent will adopt, such as intergenerational parenting styles, advice from a relative, community member or professional person. The current study suggests that a good support system from a partner, family, and/ the community enable effective parenting within the first 1000 days. Similarly, support received from and access to partners, families, friends and community members have been reported as enablers for effective parenting [13].. This support may include friendship networks and the experience and wisdom of other parents. This will aid in shaping parenting knowledge, skills and practices. In addition, the support from the community may provide and include emotional support and practical parenting skills needed in rearing and nurturing children and early childhood development. The experience and wisdom from other parents will serve as a source of information and enable parents to deal with unfamiliar or problematic parenting situations, particularly when parenting children with physical, mental or cognitive disabilities, or disorders [42, 45, 46]. Parenting children with disabilities and disorders may cause parents to feel overwhelmed by their roles and responsibilities, and this may affect their parenting practices [42, 47]. Thus, support from partners, relatives, friends and the communities is crucial in effective parenting practices. Parents’ behaviour has a considerable impact on the child. The effect of smoking and consuming alcohol during pregnancy and after birth has serious health implications for both mother and child and may be associated with the acquisition of cognitive and behavioral development skills [48, 49]. Therefore, parents’ healthy behaviour is important for effective parenting as everyday behaviors, particularly healthy lifestyles, may influence maternal and child mental health and well-being [50, 51]. In addition, the mental health of parents and caregivers may significantly affect the quality and dynamic of the parent–child relationship, [52] thereby enhancing or hindering effective parenting practices and responsive caregiving. Similarly, the parents' living environment is noteworthy, that is, living in a stable and supportive home with less stressful conditions and secure housing is conducive to effective parenting [13]. Conversely, stressful living circumstances such as violence (intimate partner, domestic or community violence), substance abuse, chronic physical health problems, homelessness, or unemployment, and lack of finances to support the family may contribute to ineffective parenting and have a negative effect on early childhood development [42, 53]. In addition, in this study, unsafe and unconducive home environments and communities have been identified as a barrier to effective parenting within the first 1000 days. This type of space discourages parents and children from connecting and socialising with others within the community. Communities can be categorised based on the presence or absence of certain infrastructures (such as housing, schools, health services, shops, public transportation, internet access, safe outdoor play spaces and equipment for children, sports and recreational facilities) and features of the physical environment (such as access to clean air, water and sanitation) [13]. Children living in unstable and disorganised home environments are at risk of adverse cognitive, social and behavioral outcomes [54, 55].

Similarly, living environment stress, low income, or unemployment has primary and secondary effects on parenting practices, the parent–child relationship, and early childhood development outcomes. Furthermore, it has an indirect influence on parents’ beliefs in terms of focusing on survival and meeting the basic needs of providing food for the family more than ensuring that children have adequate opportunities to achieve development outcomes [42, 53]. In dire situations which have led to families living in poverty, poverty affects the entire family functioning, including parenting and its various dimensions (such as supervision, monitoring and consistency in the use of such discipline) [56, 57]. Less affluent are less likely to afford quality child care which may have an indirect impact on child safety and their children may receive lower-quality of care [58]. Moreover, children who are raised in poverty are at greater risk of poor developmental outcomes [59].

Moreover, when both parents are employed, it may not be beneficial to their children because working long hours or having a stressful job can prevent parents from being physically and emotionally present to create a parent–child bond.[58]. However, an income enables parents to independently provide for and improve their children’s lives [58]. Therefore, considering the benefits of having an income and being employed, this may be considered as a facilitator of effective parenting.

Research has found that high parental education and knowledge levels have a positive and precautious influence on the parent–child relationship and early childhood development outcomes [44, 60, 61]. Knowledge of children's development, children's needs, parenting practices, services, and support systems are important for effective parenting [62]. It has been reported that parents who are knowledgeable about child development are more likely to have quality parent–child interactions and demonstrate supportive and effective parenting practices. [62]. In addition, parents who know about specific evidence-based parenting practices are more likely to engage in these practices, be more responsive and change their behaviour at each age and stage of the child [42, 63–65].

### Implications of the study

This study provided insight into the enablers and barriers to effective parenting within the first 1000 days of life. The study findings are important to improve parent–child relationships and child developmental outcomes, particularly in developing countries. This exploration could facilitate the development of policies, interventions and locally based programmes to address the barriers and highlight the factors to effective parenting within the first 1000 days of life. In addition to the development of policies, interventions and programmes, promoting effective parenting will assist the government towards the realisation of Sustainable Development Goals, particularly goals one, two, three, four and ten.

### Recommendations of the study

There is a need to research how to promote effective parenting within the first 1000 days. Also, research needs to explore how to remove barriers to effective parenting within the first 1000 days. The government needs to revise and develop policies and interventions to promote effective parenting. The government and service providers should create empowerment programmes to empower the parents so that parents can provide for their children. Service providers should create awareness programmes about the available services within the communities to assist parents in raising their children. Parents should be trained on parenting skills and they should be exposed to parenting programmes.

### Strengths and limitations of the study

One of the strengths of the study is that a qualitative method with an explorative research design was used to collect data. The research design used allowed an exploration of the phenomenon under study. Another strength of the study is that the perspectives of the participants who are directly affected by the phenomenon under study were explored. Parents who had children between 0 and 2 years of age were interviewed. One of the limitations of this study is that all the respondents were female, except one. Another limitation is that we did not explore the perspectives of all stakeholders (government officials, service providers and community leaders) responsible for the child’s holistic development within the first 1000 days.

## Conclusions

Enablers that need to be promoted for effective parenting within 1000 days of life include a support system, healthy behaviours/environment, unemployment/job opportunities, religion, information/knowledge, and professional assistance for parents. Also, barriers that needed to be removed for effective parenting were low socio-economic circumstances, environmental circumstances, lack of partner’s support, the negative impact of technology, and lack of access to services. This is because effective parenting is vital in improving developmental outcomes for children within the first 1000 days of life. Therefore, there is a need to develop policies and interventions to promote effective parenting within the first 1000 days in the communities. The development of policies and interventions to promote effective parenting will enable children to survive, thrive and transform their human potentials.

## Data Availability

The datasets used and/or analysed during the current study are available from the corresponding author on reasonable request.

## Abbreviations

MNCH: Maternal Newborn and Child Health
SDGs: Sustainable Development Goals
UNICEF: United Nations Children’s Emergency Fund
WHO: World Health Organisation
COREQ: Criteria for Reporting Qualitative Research
NRF: National Research Foundation
